# Real-time fMRI neurofeedback modulates auditory cortex activity and connectivity in schizophrenia patients with auditory hallucinations: A controlled study

**DOI:** 10.1016/j.pscychresns.2025.112050

**Published:** 2025-08-22

**Authors:** Clemens C.C. Bauer, Jiahe Zhang, Francesca Morfini, Oliver Hinds, Paul Wighton, Yoonji Lee, Lena Stone, Angelina Awad, Kana Okano, Melissa Hwang, Jude Hammoud, Paul Nestor, Susan Whitfield-Gabrieli, Ann K. Shinn, Margaret A. Niznikiewicz

**Affiliations:** a Department of Psychology, Northeastern University, Boston, MA 02115, USA; b Department of Brain and Cognitive Sciences and McGovern Institute for Brain Research, Massachusetts Institute of Technology, Cambridge, MA 02139, USA; c Department of Psychiatry, Harvard Medical School, Boston, MA 02115, USA; d Center for Precision Psychiatry, Massachusetts General Hospital, Boston, MA, 02114, USA; e Orchard Scientific, Yucca Valley, CA, USA; f Athinoula A. Martinos Center for Biomedical Imaging, Massachusetts General Hospital, Charlestown, MA, USA; g Psychotic Disorders Division, McLean Hospital, Belmont, MA 02478, USA; h Laboratory of Neuroscience, Boston VA Healthcare System, Boston, MA, 02130, USA; i Boston VA Healthcare System, Boston, MA 02130

**Keywords:** Superior temporal gyrus, Default mode network, Mindfulness, Dorsolateral prefrontal cortex, Functional magnetic resonance imaging

## Abstract

**Background and Hypothesis:**

Auditory hallucinations (AHs) affect 60–80 % of schizophrenia patients and often resist antipsychotic treatment. AHs involve superior temporal gyrus (STG) hyperactivity and disrupted auditory-cognitive control connectivity. Real-time fMRI neurofeedback (NFB) enables voluntary modulation of targeted brain regions. We previously showed STG-targeted NFB with mindfulness meditation reduced STG activation and AHs in one session. However, whether effects are specific to hallucination-related regions versus placebo, and whether NFB modulates broader networks, remained unclear.

**Study Design:**

This randomized, sham-controlled trial examined NFB specificity and network effects. Twenty-three adults with schizophrenia/schizoaffective disorder and medication-resistant hallucinations practiced mindfulness meditation while receiving neurofeedback from either STG (*n* = 10, Real-NFB) or motor cortex (*n* = 13, Sham-NFB control). Sham participants subsequently received Real-NFB, providing within-subject comparison.

**Study Results:**

Both groups showed reduced AHs post-NFB without group differences. However, compared to Sham-NFB, Real-NFB produced greater reductions in secondary auditory cortex activation and connectivity between auditory cortex and cognitive control regions (dorsolateral prefrontal cortex and anterior cingulate). These connectivity reductions persisted in the Real-after-Sham condition. Both groups showed reduced primary auditory cortex activation, suggesting mindfulness meditation independently regulates bottom-up hallucination processes.

**Conclusions:**

Region-specific NFB targeting produces distinct neural changes beyond symptom reduction. STG-targeted NFB differentially modulates auditory-cognitive control networks, potentially restoring the disrupted balance between bottom-up sensory processing and top-down control in AHs. These findings highlight the importance of anatomically-informed NFB targets and provide mechanistic insights for developing precision interventions for treatment-resistant psychiatric symptoms.

## 1. Introduction

Auditory verbal hallucinations (AHs) are a hallmark symptom of schizophrenia (SZ), experienced by approximately 60–80 % of individuals with the disorder. (Kråkvik et al., 2015; Lim et al., 2016) AHs typically manifest as hearing voices or sounds in the absence of any external auditory stimulus and are often distressing and intrusive. The neural substrates underlying AHs in SZ involve distributed brain regions forming functional brain networks. Hyperactivity in the superior temporal gyrus (STG), the anatomical location of the auditory cortex (AC), has been implicated in the occurrence of AHs. (Kaur et al., 2020; Moseley et al., 2014; Hugdahl et al., 2008; Hugdahl, 2015; Tracy and Shergill, 2013) According to Northoff’s resting-state hypothesis of AHs, STG hyperactivity coexists with abnormal STG interactions with the Default Mode Network (DMN) and the Central Executive Network (CEN). (Shao et al., 2021; Northoff and Qin, 2011; Northoff, 2015) Because the DMN is normally active during rest and involved in self-referential thoughts while the CEN is associated with other cognitive functions (e.g. working memory), (Buckner and DiNicola, 2019; Whitfield-Gabrieli and Ford, 2012; Raichle et al., 2001) AHs may result from a complex interaction of abnormally elevated resting state activity in the STG, its hyperconnectivity with the DMN, and deficient regulation from CEN that normally enables the distinction between internally-generated and externally-induced activity. (Northoff, 2015; Makino, 2019) According to this model, a combination of hyperactivity and hyperconnectivity within and between AC and self-referential DMN in the absence of external stimuli are misidentified and perceived as AHs. Northoff’s hypothesis extends earlier hypotheses that proposed misattribution of self-generated stimuli to others instead of self, (Fu et al., 2008; Frith and Done, 1988) as well as models that suggest a breakdown in the network of brain regions involved in auditory processing and inhibitory control. (Hugdahl, 2015)

AHs can be resistant, or only partially responsive, to antipsychotic medications, (Shergill et al., 1998; Gillespie et al., 2017; Pandey and Kalita, 2022) which points to the need for alternative therapeutic strategies. A promising new, non-invasive approach is real-time neurofeedback, a procedure in which individuals receive moment-by-moment feedback based on their brain signals [e.g., electroencephalography, magnetoencephalography, functional magnetic resonance imaging (fMRI)] (Sitaram et al., 2017). fMRI-based real-time neurofeedback (NFB), in particular, has been promising for its ability to target specific brain regions with high spatial precision as well as to monitor multiple brain regions simultaneously, thereby providing a more comprehensive and personalized approach to brain regulation and symptom management. (Fede et al., 2020; Pindi et al., February 2021) There has been encouraging progress investigating the efficacy of NFB as a treatment for AHs. (Dyck et al., 2016; Humpston et al., 2020; Orlov et al., 2018; Hirano and Tamura, 2021) However, most studies do not provide overt instructions or strategies to help subjects successfully regulate fMRI signals, resulting in the need for multiple training sessions for each participant. (Sitaram et al., 2017; Haugg et al., 2021) Clear guidance for participants with SZ is critical as this population exhibits self-regulation deficits including impaired cognitive control and emotion regulation. (O’Driscoll et al., 2014) These impairments affect decision-making and reinforcement learning, (Culbreth et al., 2016; Santosh et al., 2015; Lesh et al., 2011) making it more challenging for individuals with schizophrenia to acquire new skills. Combining mindfulness meditation and NFB, we recently developed an innovative paradigm whereby patients with treatment resistant AHs learn to modulate brain activation in the STG. (Okano et al., 2020)

Specifically, we taught participants “mental-noting” meditation to help them ignore pre-recorded sentences spoken in a stranger’s voice and downregulate STG activation. This guided neurofeedback training resulted in reduced STG activation and lower AHs scores after a single 21-minute session, suggesting this combined approach may be effective for medication-resistant AHs. (Okano et al., 2020) Our previous study had limitations, notably the absence of a sham condition and comprehensive assessment of whole-brain impact. To address these gaps, the current sham-controlled, randomized study aims to investigate the broader brain network effects of NFB. Here we sought to examine if NFB delivered from a brain region implicated in AHs (STG) would engage cognitive control brain regions in contrast to NFB from an unrelated area. Additionally, we aimed to replicate our previous findings showing that NFB reduces AHs and primary cortex activation. (Okano et al., 2020) The current study has been registered with clinicaltrials.gov, updated May 17, 2024 (trial identifier: NCT05299749).

## 2. Methods

### 2.1. Overview of study design

**Fig. 1.**
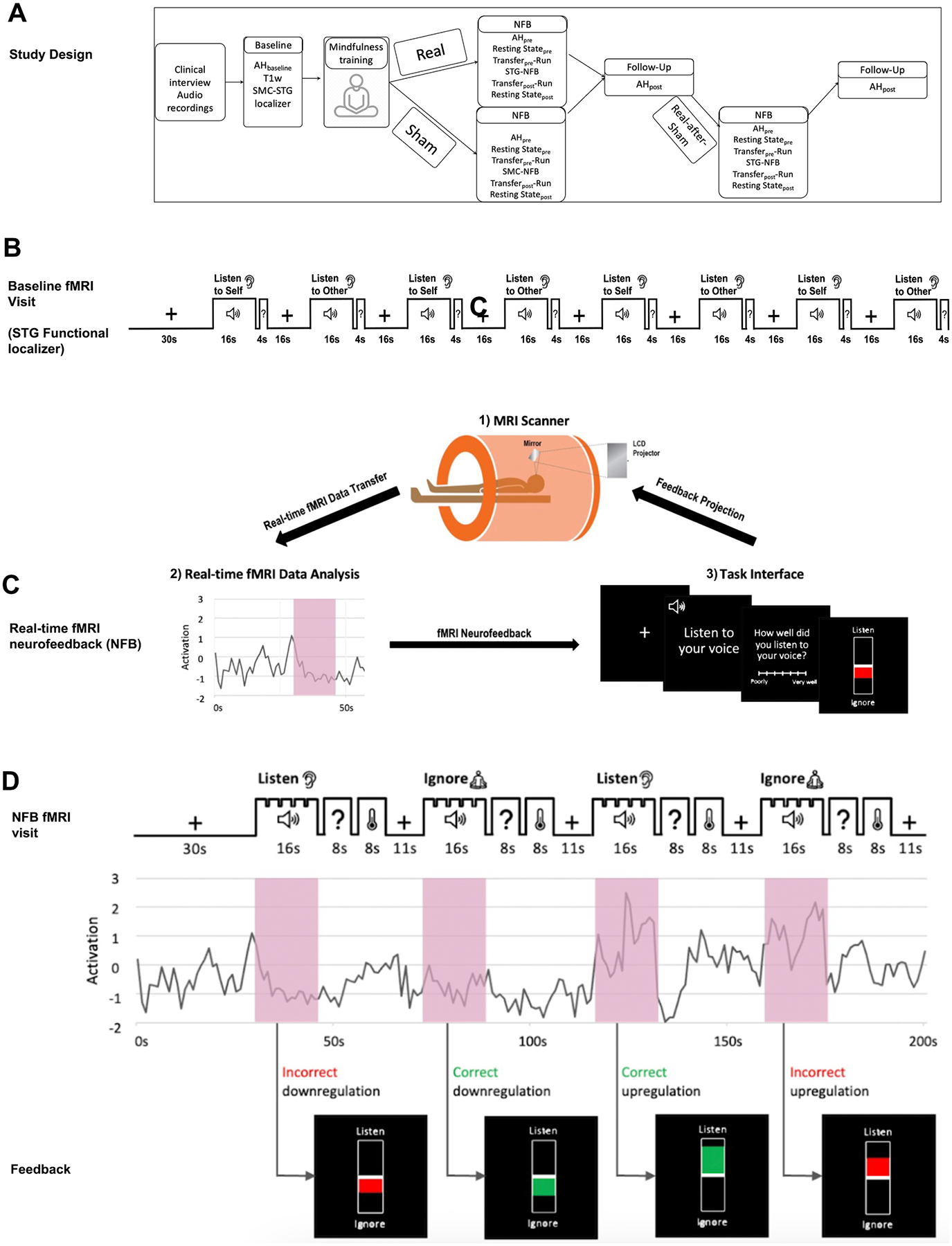
STG targeting, real-time fMRI neurofeedback experimental design (NFB). A) Overall study design. B) Baseline fMRI visit with STG functional localizer. C) Real-time fMRI neurofeedback setup that includes 1) the magnetic resonance imaging scanner, 2) real-time data analysis and 3) task Interface that forms a closed loop of information flow to the participant. D) NFB task design with an example of fMRI activation time series of the target region (superior temporal gyrus (STG) for Real-NFB and motor (MC) for Sham-NFB) which illustrates four possible types of neurofeedback that a participant could receive during the neurofeedback regulation blocks (red shadow). Green color on the thermometer indicates modulation in the correct direction (i.e., downregulation during ‘Ignore’ condition or upregulation during ‘Listen’ condition) and red color indicates modulation in the wrong direction (i.e., downregulation during ‘Listen’ condition or upregulation during ‘Ignore’ condition).

Data were acquired across multiple visits: initial clinical assessment, baseline fMRI localization, two NFB visits (data related to the second NFB for the Real-NFB group are not included here), and clinical follow-up (Fig. 1A). Participants were randomly assigned to Real-NFB or Sham-NFB groups using simple randomization (coin flip) for the first participant, then alternating group assignments for subsequent participants to ensure balanced groups. Initial visits included demographic collection, clinical and neurocognitive assessments, and voice recordings for subsequent fMRI sessions. Baseline fMRI comprised a T1-weighted structural scan, an STG-localizer task identifying regions sensitive to self-other voice distinction, and a resting state scan for motor cortex localization.

During the first neurofeedback visit, the Real-NFB group received feedback based on STG activation, while the Sham-NFB group received motor cortex feedback. The Sham-NFB group later received STG-based feedback in a second session (Real-after-Sham-NFB condition). Before and after neurofeedback, participants completed transfer tasks without feedback. Participants remained blinded to the feedback source (STG for Real-NFB and Real-after-Sham-NFB, motor cortex for Sham-NFB). All procedures were identical except for the region generating the feedback signal.

### 2.2. Participants

Participants were recruited from clinical services at the Boston Veterans Affairs Healthcare System and McLean Hospital. All participants gave written informed consent obtained in accordance with the guidelines of the Institutional Review Board (IRB) of Boston VA Healthcare System and the IRB of Harvard Medical School, which served as the IRB of record for McLean Hospital, Massachusetts Institute of Technology (MIT), and Northeastern University (see Supplementary Methods for detailed eligibility criteria). Forty-one patients enrolled in this study. After enrollment, six participants were deemed ineligible due to meeting exclusion criteria. Five participants withdrew during the course of the study. Five participants were lost to follow-up. This resulted in a total of 25 participants (36.1 ± 10.0 years; 24–54 years; 24 % females) who were randomly assigned to receive either Real-NFB (*n* = 12) or Sham-NFB (*n* = 13). After randomization, two participants in the Sham-NFB group were lost to follow-up. Two Real-NFB participants had incomplete transfer task fMRI data after NFB. One participant’s AHs score was completed >200 days after the NFB session due to COVID lockdown, therefore we excluded the post-NFB AHs scores. See the CONSORT diagram (Figure S1) for details.

Sociodemographic and clinical characteristics are summarized in Table 1. A sample of 23 participants (mean age=36.34 ± 10.26; 25 % females) was included for the final analysis Real-NFB (*n* = 10) or Sham-NFB (*n* = 13). During the course of our study, we were required to change sites and scanners. Among the 23 participants, six were scanned on a Siemens Trio scanner at MIT, 10 were scanned on a Siemens Prisma scanner at MIT and seven were scanned on a Siemens Prisma scanner at Northeastern University (for details see Table S1).

For analyses comparing Real-after-Sham-NFB to Sham-NFB conditions, we retained the full Sham-NFB sample (*n* = 13) rather than restricting to only the 11 participants who completed both sessions, thereby maximizing statistical power while acknowledging this introduces some between-subject variability.

### 2.3. Clinical assessments

All assessments were conducted by trained clinicians blind to group assignment. We administered the SCID-5 to verify diagnoses of schizophrenia or schizoaffective disorder. To measure the severity of AHs, which was our primary symptom of interest, we administered the auditory hallucinations subscale of the Psychotic Symptom Rating Scale (PSYRATS-AHs (Haddock et al., 1999)). We administered the PSYRATS-AH at the baseline clinical visit, at the beginning of every fMRI study visit, and also at the clinical follow-up visit. All baseline clinical and neuropsychological evaluations were conducted in-person.

### 2.4. Audio recording and processing

Patients recorded 170 sentences to be used in the ‘self-voice’ condition on an Olympus, digital recorder, model VN-722 PC. Recording was carried out in a sound-proof room and sentences were read without emotional intonation. All sentences had a structure of subject + verb + adjective + object (e.g., “Jane liked chocolate chip cookies”) with an average length of 6.65 words (SD=1.13). All sentences were neutral in affect and written in third person. Recorded sentences were processed in 3 steps: 1. Audio intensity was normalized using Praat (http://www.fon.hum.uva.nl/praat/). 2. Background noise was removed using Audacity (http://www.audacityteam.org). 3. Individual sentences were segmented using Praat. The same sentences were recorded by a male in his 40 s and edited in the same manner to be used in the ‘male other’ condition. We further processed the ‘male other’ sentence in two ways to mimic female voice: 1) We changed the formant shift ratio to 1.2 to account for a shorter vocal tract. 2 We changed the pitch median to 220 Hz to account for greater vocal fold tension.

### 2.5. Superior temporal gyrus (STG) functional localizer

Participant-specific STG was defined during the baseline visit using a functional localizer task (Self-Other Task; Fig. 1B) in which participants listened to pre-recorded sentences. Eighty unique sentences were presented: 40 sentences in the subject’s own voice and 40 in a sex-matched stranger’s voice. The Self-Other Task included four blocks each of 16 s duration (i.e., two for self-voice, two for other-voice) interspersed with fixation blocks of the same length. Each ‘self-voice’ block included five unique sentences spoken in the subject’s voice; each ‘other-voice’ block included five unique sentences spoken in a sex-matched stranger’s voice. For each subject, sentences in self-voice and other-voice did not overlap. The fixation blocks included a silent block where participants gazed at a crosshair in the center of the screen. To help sustain attention, after each voiced block, subjects were prompted with a ‘yes’ or ‘no’ question on the screen regarding the content of the last sentence they had heard in the block (e.g., “Did Jane like chocolate chip cookies?”) and submitted their response via button press. After the fixation block, they answered a real-world question (e.g., “Is the White House in Washington D.C.?”). These blocks were split evenly across two functional runs (4 min and 26 s each). Each run contained 12 pseudo randomized blocks (4 blocks per condition) such that fixation blocks did not appear back to back. Using offline analysis after data collection, we created participant-specific target regions of interest (ROIs) by: 1) Contrasting self-voice > other-voice activation 2) Identifying significant clusters in bilateral STG 3) Selecting the top 100 most active voxels as the final ROI.

### 2.6. Motor cortex (MC) functional localizer

We identified each participant’s MC for the sham neurofeedback condition using resting-state fMRI data. The localization process involved several steps: Data Preprocessing: Using FSL 6.0^34^ we preprocessed the resting-state data with standard procedures: motion correction, brain extraction, registration to standard space, spatial smoothing, and bandpass filtering to remove noise (detailed parameters in (CC Bauer et al., 2020)). Component Identification: We used Independent Component Analysis (ICA) (Beckmann et al., 2005) to decompose each participant’s brain activity into 35 distinct spatial patterns. (Jenkinson et al., 2012) To identify which pattern corresponded to the motor cortex, we compared each component to a reference motor cortex map derived from ~1000 participants. (Yeo et al., 2011) MC Mask Creation: For each participant, we: Selected the component with the highest correlation to the reference MC map Retained only the most active voxels (top 10 %) within this component Created a binary mask defining their individual motor cortex region Verified that each mask appropriately covered expected motor regions. (Franco et al., 2009) This approach ensured that sham neurofeedback was delivered from a functionally-defined motor cortex region specific to each participant, providing a control condition unrelated to auditory hallucinations. For details on MRI Data Acquisition see supplemental material and Table S1.

### 2.7. Mental-noting training

We trained participants on a mindfulness technique called “mental-noting” (from Vipassana insight meditation (Sayadaw, 2014)) which consists of two main components: “concentration” and “observing sensory experience”. The 15-minute training occurred outside the scanner, immediately after baseline PSYRATS assessment and before MRI scanning.

### 2.8. Training protocol

#### Phase 1 - Introduction and Basic Practice (5 min):

Participants learned that mental-noting involves labeling present-moment sensory experiences (“hearing,” “seeing,” “feeling”) without engaging with content details. They selected a personal “anchor” (e.g., breath, toe sensations) to redirect attention when stuck on repetitive sensations. After the experimenter demonstrated by verbalizing sensory awareness approximately once per second, participants practiced mental noting aloud to ensure proper technique.

#### Phase 2 - Distraction Practice (5–7 min):

Participants maintained mental-noting while listening to recorded stories. The goal was to continue noting sensory experiences while allowing story content to “fade away” without processing. After each story, participants recalled details to assess their level of engagement.

#### Phase 3 - Performance Criterion (3–5 min):

Training continued until participants demonstrated successful disengagement by recalling <50 % of story details on two consecutive trials. This criterion ensured participants could effectively use mental-noting to ignore auditory content.

All participants achieved criterion within the 15-minute period. They were then instructed to use this mental-noting strategy during “ignore-voice” blocks in the scanner to help them disregard all sounds, including the presented voices (see Supplementary Methods for detailed training script).

### 2.9. Real-NFB task

NFB was delivered in the form of a thermometer with the color green representing positive-feedback and the color red representing negative-feedback. The extent of the thermometer represented brain activation. NFB setup includes three main components that form a loop of information flow (Fig. 1C). All participants randomized to Real-NFB (*n* = 10) heard a set of 80 unique sentences (average length=6.64 words, SD=1.31) (a different set of sentences than the ones presented during the localizer scan) presented via earphones in the scanner. Forty sentences were in their own voice and 40 in a stranger’s voice. These sentences were not significantly different in their length from the 80 sentences heard in the STG localizer Self-Other Task [*t*(158)=0.34, *p*=.73]. There were six runs for this task, each lasting 2.5 min. The first and last runs were transfer runs (i.e., they did not provide NFB; NFB-transfer-pre and -post), while runs 2–5 provided real-time NFB from individualized STG masks. Each run had four randomized blocks composed of two listen blocks and two ignore blocks, each lasting 16 s (Fig. 1D). Before each block, participants saw a prompt stating either “listen to self” to signal that they were asked to attend to the sentences played over the earphones (resulting in up-regulating STG) or “ignore all sounds”, to signal that they were asked to ignore the sentences along with any other environmental noises including the scanner noise (resulting in down-regulating STG). To successfully ignore the other-voice sentences, participants were instructed to use the mental-noting strategy. After each feedback block, participants saw a ‘thermometer’ showing their activation level in the STG. The height of the ‘thermometer’ bar reflected a median activity level for a given block. In addition, to gauge the participant’s assessment of their own performance during the tasks, after each listen block, a prompt asked the participant to rate how well they were able to attend to the voices on a scale of 1 (completely attended to) to 6 (could not attend at all); after each ignore block, a similar prompt asked how well they were able to ignore all sounds. Participants responded to these prompts via button press. The STG estimates were calculated as the median activity across all voxels within each participant’s STG mask (as defined by STG Functional Localizer) and co-registered to the current fMRI volumes. To accomplish the voxel-wise estimation in real-time, (Hinds et al., 2011) we first collected 30 s of baseline data and then continuously performed an in-cremental general linear model (GLM) fit with subsequent incoming images. This method accounts for the mean voxel signal and linear trends. To discount components of the voxel signal due to nuisance sources (e.g., low-frequency signal drifts), the GLM reconstruction of the expected voxel intensity at time t was subtracted from the measured voxel intensity at time t, leaving a residual signal that has components due to two sources: BOLD signal fluctuations and unmodeled fMRI noise. This residual was scaled by an estimate of voxel reliability, which was computed as the average GLM residual over the first 25 functional images of the baseline. This analysis resulted in an estimate of the strength of activation at each voxel at time t in units of standard deviation.

### 2.10. Sham-NFB task

All participants randomized to Sham-NFB (*n* = 13) completed the feedback task that was structured identically to the Real-NFB with the exception that feedback was provided from the personalized MC. All other instructions and parameters were the same.

### 2.11. fMRI data preprocessing for mask generation and analysis

Preprocessing and data analyses were completed using FSL Version 6.0.3. (Jenkinson et al., 2012) Preprocessing included brain extraction using BET (Brain Extraction Tool), motion correction using MCFLIRT (Motion Correction FMRIB’s Linear Image Registration Tool). (Smith et al., 2004) The remaining fMRI signals were spatially blurred with 6-mm full-width-at-half-maximum (FWHM) Gaussian kernel. A subject-dependent number of individual nuisance regressors for removing outlier time points were created using the fsl_motion_outliers tool, employing the DVARS metric. This metric applies a threshold on the root-mean-squared intensity difference of volume N to volume *N* + 1. (Power et al., 2012) The threshold used was the standard boxplot outlier threshold set in FSL. (Power et al., 2012) Participants were excluded if they had >20 % of movement outliers. (Jones et al., 2023)

### 2.12. Clinical analysis, effect of NFB on psyrats-ah scores

Statistical tests for PSYRATS-AH data were conducted using R Studio Version 1.0.136. (RDC, 2008). Statistical significance level was set at 0.05.

#### 2.12.1. Brain activation analyses

Activation analyses were restricted to the bilateral anterior and posterior temporal lobes as defined by the Harvard-Oxford atlas (Desikan et al., 2006) with a threshold at 85 % probability to remove any non temporal lobe voxels as an *a priori* ROI based on a previous report. (Okano et al., 2020) We examined how temporal lobe activation changes after NFB (pre-post) differed between Real-NFB and Sham-NFB while participants were using the nothing practice to ignore the stranger’s voice (other-voice>fixation-block, hereafter ‘ignore-other-voice’).

### 2.13. Functional connectivity analyses

To examine functional connectivity in Real-NFB relative to Sham-NFB, we performed a generalized psychophysiological interaction (gPPI) analysis. (Friston et al., 1997; McLaren et al., 2012) Specifically, we examined the functional connectivity of a secondary auditory cortex (AC) cluster identified from the activation analysis during the *ignore-voice* blocks (depicted in Blue in Fig. 3A, peak: *X* = 56, *Y*= −24, *Z*= −6, BA 22). First-level GLM analyses included four regressors: psychological, physiological, gPPI, and nuisance. *Listen-voice-* and *ignore-voice*-*block* durations comprised the psychological regressor, modeled as a boxcar function, convolved with a single gamma hemodynamic response function. For each participant, we extracted the physiological regressor as the time series for the functional cluster identified in the two-way mixed effect ANOVA during the *ignore-voice-block* contrast across groups. The gPPI regressor was the product of the demeaned physiological regressor and the psychological regressor, which was zero-centered between the minimum and maximum values. The following nuisance regressors were modeled: global mean time series of each preprocessed run, six motion parameters, and motion outliers. These individual contrast images were then entered into a second level fixed-effects analysis including both runs for each participant. Mixed-effect analysis of variance tests with factors of Time (pre-NFB vs. post-NFB) x Group (Real-NFB vs. Sham-NFB) on each participant’s STG and whole brain connectivity Z scores were conducted. Significant main effects and interactions were followed up with post hoc testing.

### 2.14. Statistical tests

For behavioral data (PSYRATS-AH scores), we conducted two-way mixed ANOVAs with factors of Time (pre vs. post) and Group (Real-NFB vs. Sham-NFB) or Condition (Real-after-Sham-NFB vs. Sham-NFB). Significant main effects were followed by post-hoc paired *t*-tests within groups. All behavioral analyses used α = 0.05 with no correction for multiple comparisons given our single primary outcome measure.

For neuroimaging data, results are reported at p-FWE<0.05 with small volume correction for bilateral temporal lobes (Harvard-Oxford atlas). (Desikan et al., 2006; Poldrack, 2007; Worsley et al., 1996) Whole-brain and gPPI analyses used FSL randomize (Winkler et al., 2014; Harrison et al., 2017) with 5000 permutations, automatic outlier deweighting, and probability threshold-free cluster enhancement (TFCE). (Spisák et al., 2019) This approach provides robust multiple comparison correction for neuroimaging data. Results are thresholded at *z* > 3.1 (*p*<.001). All post-hoc neuroimaging comparisons were conducted within the same statistical framework to maintain appropriate error control.

### 2.15. Replication analyses

We sought to replicate our previous finding (Okano et al., 2020) of reduced primary auditory cortex activation (MNI:60, −18,10; BA41), post-neurofeedback, using a 10 mm sphere ROI. Self-voice vs. other-voice contrasts were calculated per run, with scanner as a nuisance regressor. We correlated hallucination scores with ROI activation during ignore-voice blocks. Details in supplement.

## 3. Results

### 3.1. Changes in psyrats-ah

**Fig. 2.**
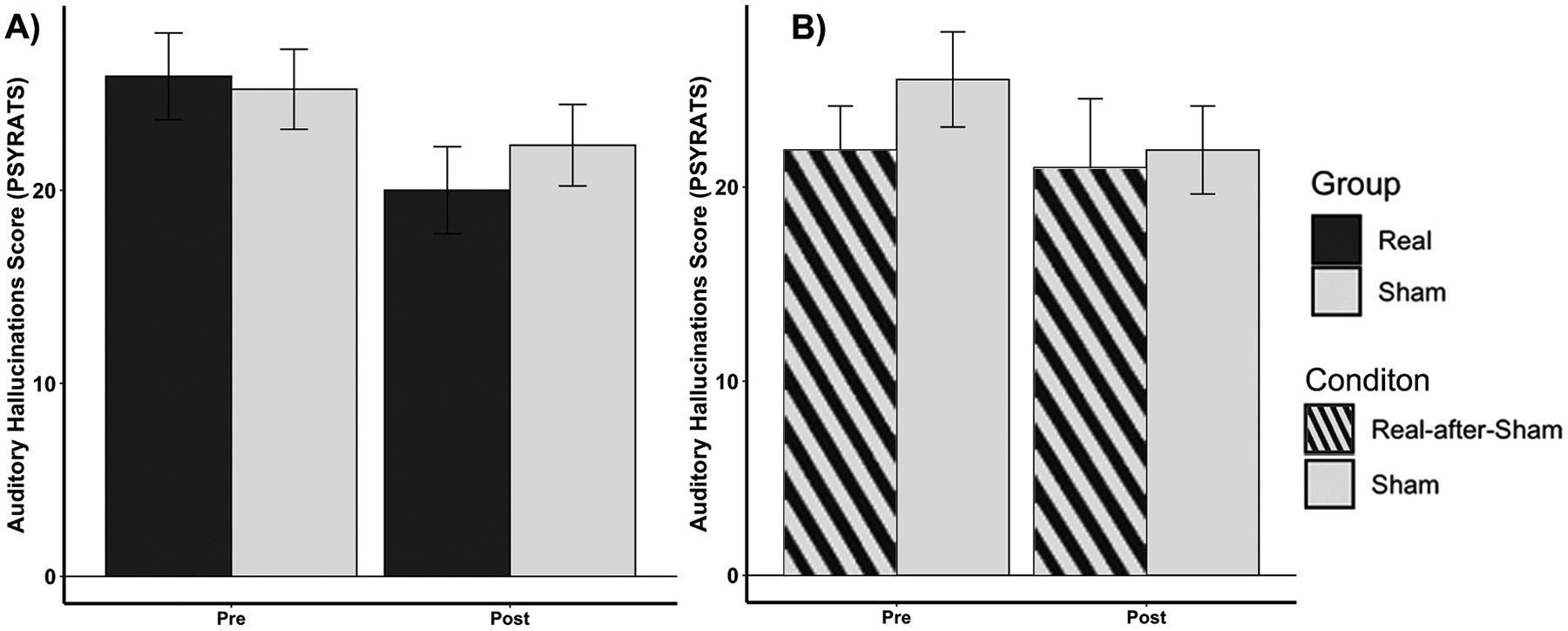
Auditory hallucinations score changes post-NFB. (A) AH-Scores pre- and post-NFB for Real-NFB (*n* = 10) and Sham-NFB (*n* = 13) groups. (B) AH-Scores pre- and post-NFB for Real-after-Sham-NFB (*n* = 11) and Sham-NFB conditions. Auditory hallucinations were assessed using the Psychotic Symptom Rating Scale (PSYRATS (Drake et al., 2007)). All bars reflect mean and all error lines reflect standard error of the mean.

Paired *t*-tests within the groups showed improvements in AHs, as measured by the PSYRATS-AH, from pre- to post-intervention for both Real-NFB [*t*(10)=1.87, *p*=.04, Cohen’s *d* = 0.82)] and Sham-NFB [*t*(10)=1.90, *p*=.04, Cohen’s *d* = 0.48]. Using a two-way ANOVA to investigate the effects of Real-NFB vs. Sham-NFB on PSYRATS-AH, we found a significant main effect of Time [F(2, 18)=2.62, *p*=.01, Cohen’s *f*^*2*^=0.42], but no effect of Group [F(2, 18)=1.33, *p*=.19] or Time x Group [F(2, 18)=1.62, *p*=.12] interaction (Fig. 2A).

Similarly, a repeated measures ANOVA comparing Real-after-Sham-NFB and Sham-NFB, showed a significant main effect of Time [*F*(2, 16)= 2.61, *p*=.01, Cohen’s *f*^*2*^=1.14], with no effect of Condition [F(2, 16)=0.92, *p*=.37] or Time X Condition [F(2, 16)=1.05, *p*=.30] interaction (Fig. 2B). No additional reductions in AHs were observed in Real-after-Sham-NFB [*t*(8)=0.96, *p*=.18, Cohen’s *d* = 0.10]. See mean and standard deviation values for pre- and post-intervention in Table S2.

### 3.2. Neuroimaging analyses

#### 3.2.1. Motion

We did not exclude any participants due to motion as no participants exceeded 20 % of high motion outliers. (Soares et al., 2021) We did not find any difference when contrasting motion between Real-NFB and Sham-NFB groups [*F*(2, 18), *p* > 3, Cohen’s *f*^*2*^<0.1] or between Real-after-Sham-NFB and Sham-NFB conditions [*F*(2, 19)<0.90, *p* > 3, Cohen’s *f*^*2*^<0.1].

#### 3.2.2. Effect of NFB on brain activation during ignore-voice

**Fig. 3.**
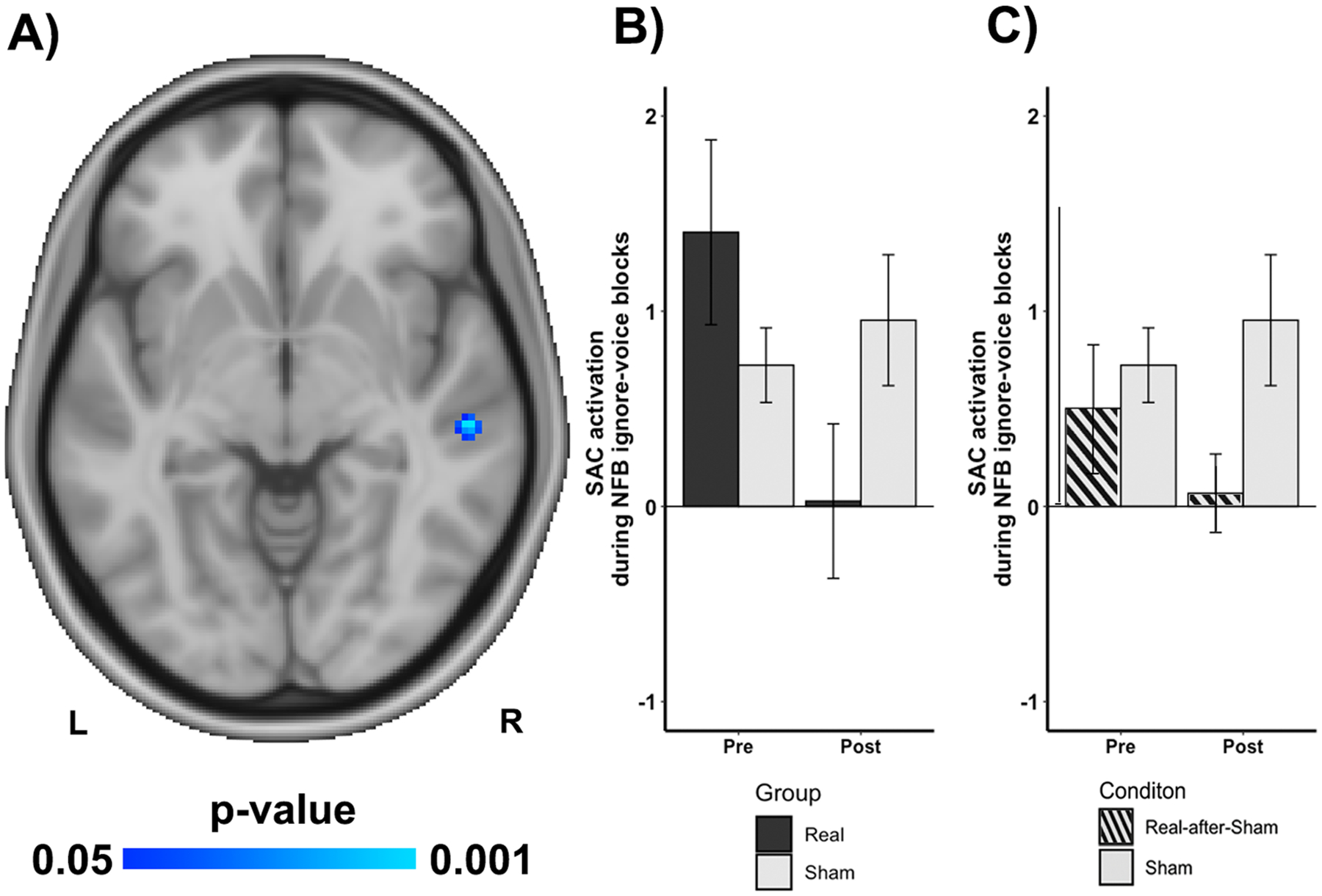
Secondary auditory cortex activation changes during, and post-NFB. (A) Region exhibiting significant Time x Group differences in secondary auditory cortex (BA22) activation during *ignore-voice* task and Real or Sham mindfulness enhanced neurofeedback (NFB). (B) Significantly decreased activation pre-to-post intervention in the Real-NFB group relative to the Sham-NFB group. (C) Significantly decreased activation pre-to-post intervention in the Real-after-Sham-NFB relative to the Sham-NFB condition. All bars reflect mean and all error lines reflect standard error of the mean; statistics are nonparametric and FWE-pTFCE small volume corrected. Secondary auditory cortex (SAC).

Using a two-way ANOVA, we tested whether brain activation in the voxels of the left and right temporal poles changed while participants were using the mental-noting practice to ignore the strangers voice. During the *ignore-voice* blocks, there was a significant Time (pre- and post-intervention) X Group (Real-NFB and Sham-NFB) interaction in the secondary AC (*n* = 23; *X* = 56, *Y*= −24, *Z*= −6, BA22; non-parametrically SVC at *p*<.001, Cohen’s *f*^*2*^=0.54; Fig. 3A), such that there was a greater reduction in right secondary AC activation after Real-NFB vs. after Sham-NFB. No main effects were found for Group or Time (*p>*.1). Post hoc two-sample *t*-tests between the groups showed no significant difference between groups at pre-NFB for right secondary AC activation during *ignore-voice* (*t*(18)=1.52, *p*=.15, Cohen’s *d* = 0.67, Fig. 3B). Paired *t*-tests within the groups only showed lower secondary AC activation for the Real-NFB group during the *ignore-voice* task in the secondary AC at post-NFB (*t*(9)=2.7, *p*=.01, Cohen’s *d* = 1.05, Fig. 3B) with no significant difference for the Sham-NFB (*t*(11)=0.78, *p*=.77, Cohen’s *d* = 0.23, Fig. 3B) or Real-after-Sham (*t*(11)=1.1, *p*= .13, Cohen’s *d* = 0.45, Fig. 3C) conditions. However, there was a trending Condition effect between Real-after-Sham-NFB and Sham-NFB (*F*(2, 19)=1.83, *p*=.08, Cohen’s *f*^*2*^=0.41; Fig. 3C), such that right secondary AC activation was further reduced in the Real-after-Sham condition following Sham-NFB. No main effects were found for Time or Condition [Fs(2, 19)<0.90, *ps*>3)]. This was confirmed by post hoc paired *t*-tests between Real-after-Sham-NFB and Sham-NFB conditions showing no significant difference between conditions at pre-NFB for secondary AC activation for *ignore-voice* [t(10)=0.52, *p*=.60, Cohen’s *d* = 0.07] but a significantly lower activation for *ignore-voice* in the Real-after-Sham-NFB than the Sham-NFB condition at Post-intervention [t(16)=2.2, *p*= .05, Cohen’s *d* = 0.74]. See mean and standard deviation values for pre- and post-intervention in Table S2.

#### 3.2.3. Effect of NFB on functional connectivity during ignore-voice

**Fig. 4.**
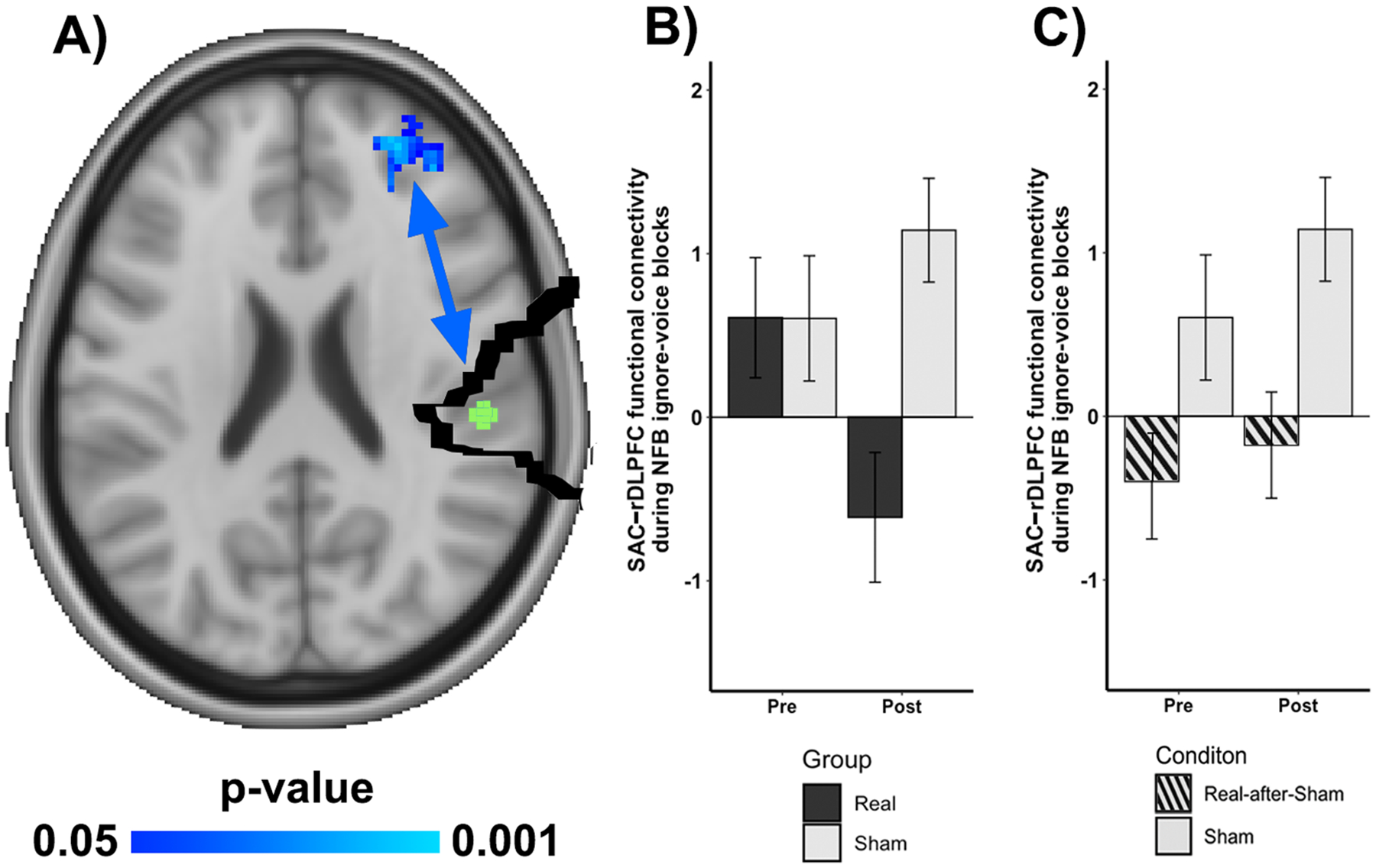
Whole brain analysis for functional connectivity changes of secondary auditory cortex during, and post-NFB. (A) Regions exhibiting significant Time x Group differences in secondary auditory cortex (SAC: green ROI seed in the superior temporal gyrus, peak: 56, 24, 6; BA22) functional connectivity and right dorsolateral prefrontal cortex during *ignore-voice* condition and Real or Sham mindfulness enhanced neurofeedback condition (NFB). (B) Significantly decreased SAC-rDLPFC functional connectivity pre-to-post intervention in the Real-NFB group relative to the Sham-NFB group. (C) Significantly decreased activation pre-to-post intervention in the Real-after-Sham-NFB condition relative to the Sham-NFB condition. All bars reflect mean and all error lines reflect standard error of the mean; statistics are nonparametric and FWE-pTFCE small volume corrected. rDLPFC=right dorsolateral prefrontal cortex; SAC=Secondary auditory cortex.

When we used gPPI analysis to examine if NFB produced changes in functional connectivity between secondary AC and the rest of the brain, we found a significant Time X Group interaction of change in functional connectivity between secondary AC and right dorsolateral prefrontal cortex (rDLPFC, *n* = 22: *X* = 28, *Y* = 50, *Z* = 22; BA10; non-parametrically at *p*<.001, Cohen’s *f*^*2*^>1.56; Fig. 4A). Functional connectivity between secondary AC and rDLPFC during *ignore-voice* reduced for the Real-NFB group while it increased for the Sham-NFB group. This was confirmed by post hoc two-sample *t*-tests between the groups showing no significant difference at pre-NFB for secondary AC functional connectivity to the rDLPFC during *ignore-voice* (*p*>.3,Cohen’s *d* < 0.1, Fig. 4B), but significantly lower secondary AC functional connectivity to the rDLPFC for *ignore-voice* in the Real-NFB group than the Sham-NFB group at post-NFB [*t*(18)=4.42, *p*=.00005, Cohen’s *d* = 1.89, Fig. 4B). Additionally, there was a significant interaction effect for Condition (*F*(2, 19)=2.25, *p*=.056, Cohen’s *f*^*2*^=1.63) of change in functional connectivity between secondary AC and rDLPFC (Fig. 4C). The pattern of this interaction suggests that functional connectivity between secondary AC and rDLPFC during *ignore-voice* remained similarly reduced after receiving Real-after-Sham-NFB, while showing a trend towards increased connectivity in the Sham-NFB condition. This was confirmed by post hoc two-sample *t*-tests between the conditions showing a trending difference at pre-NFB for secondary AC functional connectivity to rDLPFC during the *ignore-voice* block (*t*(16)=1.72, *p*=.09, Cohen’s *d* = 0.67, Fig. 4C), but a significantly lower functional connectivity for *ignore-voice* in the Real-NFB condition than in the Sham-NFB condition at Post-intervention(*t*(16)=2.74, *p*=.01, Cohen’s *d* =1.07, Fig. 4C). See mean and standard deviation values for pre- and post-intervention in Table S2.

#### 3.2.4. Effect of NFB on activation in cognitive control regions during ignore-voice

**Fig. 5.**
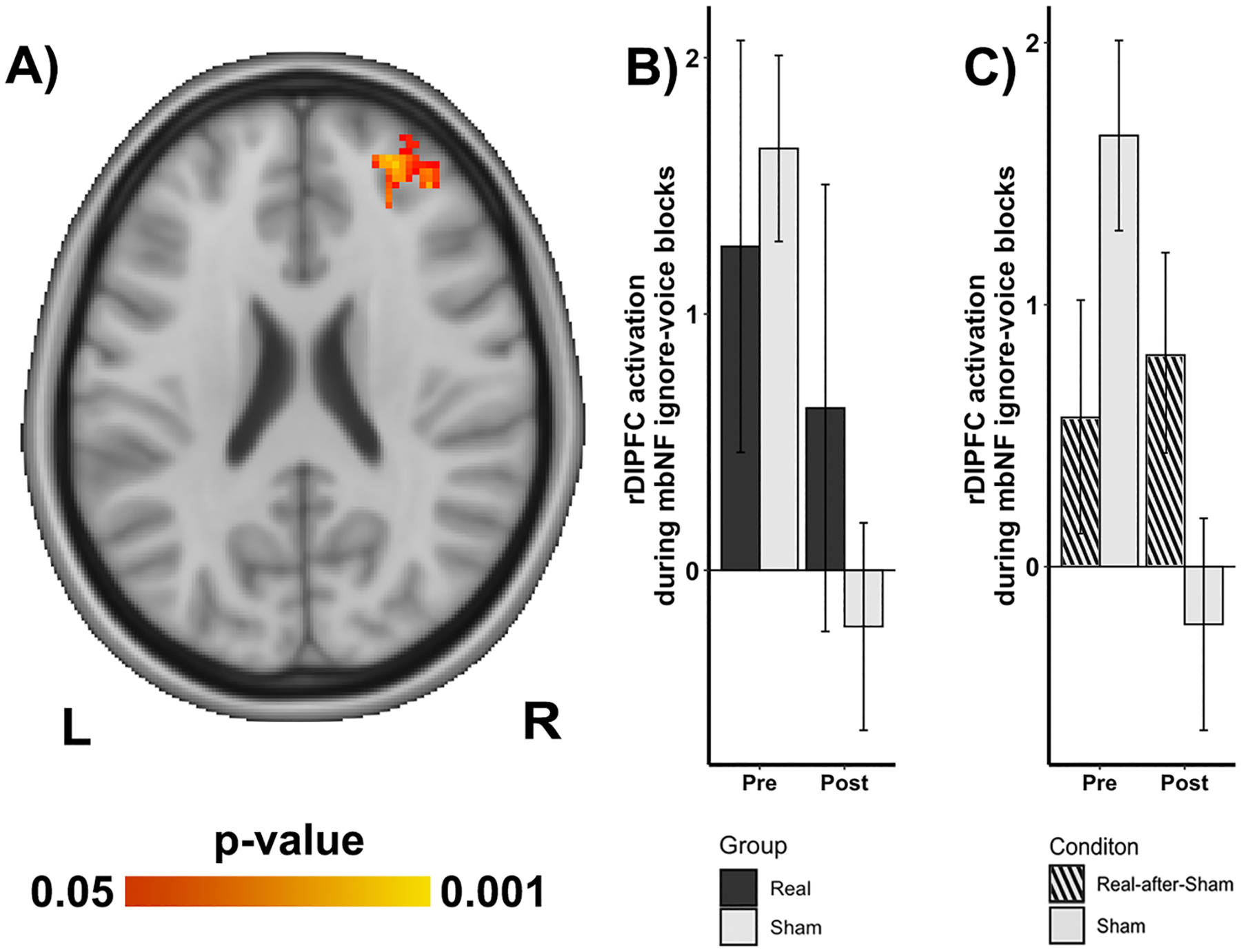
Activation changes in cognitive control brain regions during, and post-NFB. (A) Region in the right dorsolateral prefrontal cortex (rDLPFC) where activation reflects a significant Time x Group interaction. (B) rDLPFC maintained similar activation pre-to-post intervention in the Real-NFB group relative to a significant decrease in the Sham-NFB group. (C) rDPFC maintained similar activation pre-to-post intervention in the Real-after-Sham-NFB condition relative to a significant decrease in the Sham-NFB condition. All bars reflect mean and all error lines reflect standard error of the mean; statistics are nonparametric and FWE-pTFCE small volume corrected.

When assessing the activation change for the rDLPFC obtained in gPPI analyses during the *ignore-voice* blocks, there was a significant Time X Group interaction [*F*(19)=2.49, *p*=.02, Cohen’s *f*^*2*^=0.93, Fig. 5A] such that activation for the rDLPFC during *ignore-voice* was maintained in the Real-NFB group while activity significantly decreased in the Sham-NFB group. This was confirmed by post hoc paired *t*-tests within the conditions showing no significant difference from pre- to post-intervention for *ignore-voice* in the Real-NFB group [*t*(9)=1.15, *p*=.13, Cohen’s *d* = 0.22, Fig. 5B] while significantly decreased in the Sham-NFB group at post-intervention [t(12)=1.19, *p*=.003, Cohen’s *d* = 1.30 Fig. 5B].

Similarly, although there was no Time X Condition interaction for the rDLPFC (F(19)=0.71, *p*=.48, Cohen’s *f*^*2*^=0.21; Fig. 5C), the activation for the rDLPFC during *ignore-voice* was maintained after receiving Real-after-Sham NFB while activity significantly decreased in the Sham-NFB condition. This was confirmed by post hoc paired *t*-tests within the conditions showing no significant difference from pre- to post-intervention for *ignore-voice* in the Real-after-Sham-NFB condition (t (10)=0.38, *p*=.64, Cohen’s *d* = 0.16, Fig. 5C). See mean and standard deviation values for pre- and post-intervention in Table S2.

## 4. Discussion

In this study, we examined if real-time-fMRI-neurofeedback (NFB) delivered from a brain region implicated in auditory verbal hallucinations (AHs), i.e., superior temporal gyrus (STG; Real-NFB), combined with “mental-noting” practice (a type of meditation), would lead to changes in activation, functional connectivity, and in AHs scores relative to a Sham-NFB delivered from an unrelated brain area, i.e., motor cortex (MC). We found that only the Real-NFB condition showed a significant reduction in 1) activation of the secondary auditory cortex (AC), 2) activation of a cognitive control region (i.e., rDLPFC), and 3) functional connectivity changes between secondary AC and rDLPFC. AHs were reduced independent of group assignment. We also found a trend-level relationship between the reduction in secondary AC-rDLPFC connectivity and reduction in AHs-scores, in the Real-NFB group only. Finally, in a set of ROI-based replication analyses (see supplemental material), we replicated that NFB reduced primary AC activation, which is in line with our previous finding. (Okano et al., 2020)

Both Real-NFB and Sham-NFB groups showed reduced auditory hallucination scores after the intervention. Since both groups practiced mental-noting during neurofeedback training (discussed below), these reductions may be attributed to meditation-related mechanisms such as increased self-awareness or expectancy effects. (Schönenberg et al., 2021; Lee and Suhr, 2020) For a detailed analysis of the relationship between primary auditory cortex and auditory hallucinations, please see our replication analysis in the supplement.

The reduction in secondary AC activation in Real-NFB represents a significant finding for understanding and treating AHs. Secondary AC (BA22), a component of Wernicke’s area, (Gourévitch et al., 2008; Bernal and Ardila, 2016) is crucial for auditory processing, (Gourévitch et al., 2008) word-form recognition and language comprehension. (Radua et al., 2018; Hugdahl, 2009) Hyperactivation of secondary AC has been reported in individuals experiencing AHs. (Alderson-Day et al., 2016; Luvsannyam et al., April 2022) While secondary AC is not typically considered a part of the DMN, functional interactions between auditory regions and DMN are linked to internally-directed cognition and autobiographical memory retrieval. (Johnson et al., 2021; Wolf et al., 2011; Northoff, 2014) DMN contributes to self-referential processes like autobiographical memory and social cognition through interactions with medial prefrontal and posterior cingulate cortices. (Wolf et al., 2011; Northoff, 2014) Dysfunction in DMN and increased secondary AC activity underlies impairments in self-referential thinking in SZ patients (Collin et al., 2021; Doucet et al., 2020; Morfini et al., 2024) and contributes to AHs. (Northoff and Qin, 2011; Northoff, 2014) Northoff’s resting state hypothesis proposes elevated auditory cortex activity and reduced top-down modulation as mechanisms for AHs. (Northoff and Qin, 2011) This hypothesis suggests a failure to distinguish intrinsic secondary AC activity from external activity. The combination of elevated intrinsic activity in secondary AC and DMN, coding inner speech, and impaired DLPFC control result in misattributing internal activity to external sources. NFB may effectively modulate activity in this region, disrupting AHs’ neural processes. This aligns with studies showing reduced auditory activation following successful AHs treatments. (Okano et al., 2020; CC Bauer et al., 2020; Slotema et al., 2014; Zhang et al., 2025)

Our finding of a reduction in secondary AC activation was coupled with sustained activation of the DLPFC in the Real-NFB condition in contrast to DLPFC deactivation in the Sham-NFB condition. The latter may represent a response to inconsistent feedback, wherein the observed feedback is not in concordance with the perceived effort expended during Sham-NFB. (Radua et al., 2018) Conversely, during feedback that is congruent with perceived effort during Real-NFB, sustained activation in the DLPFC may regulate the hyperactivity in auditory cortices, (Hugdahl, 2015; Hugdahl, 2009) thereby restoring normal self-monitoring processes (Johnson et al., 2021; Yang et al., 2020) and reducing AHs. (Alderson-Day et al., 2016; Luvsannyam et al., April 2022)

Reduced functional connectivity between the secondary AC and rDLPFC observed in the Real-NFB condition has further implications for understanding and treating AHs. It has been proposed that abnormally increased connectivity between speech-related network (including secondary AC) (Gourévitch et al., 2008; Bernal and Ardila, 2016) and executive network (including DLPFC) could reflect increased attentional resources for salient internal events (e.g., AHs) and inefficient top–down suppression. (Wolf et al., 2011) Prior studies have provided evidence of increased connectivity between the secondary AC and the central executive network (CEN) in individuals experiencing AHs. (Alderson-Day et al., 2016; Alderson-Day et al., 2016; Northoff, 2014) Our finding of decreased connectivity between secondary AC and DLPFC after Real-NFB further supports the role of brain regions involved in auditory processing and executive control play in improving AHs. (Whitfield-Gabrieli and Ford, 2012; Humpston et al., 2020; CC Bauer et al., 2020) This change in functional connectivity could influence AHs through several potential mechanisms. Previous research, such as that by Wolf et al., (Wolf et al., 2011) has demonstrated that hyperconnectivity between frontal and temporal cortices in individuals experiencing AHs may reflect increased attentional resources for salient internal events, coupled with inefficient top-down suppression and executive control. (Dyck et al., 2016; Humpston et al., 2020; CC Bauer et al., 2020) Reduced STG-rDLPFC connectivity may disrupt the abnormal neural patterns underlying AHs, potentially by diminishing the attentional resources allocated to internal auditory events. (Humpston et al., 2020; Okano et al., 2020; CC Bauer et al., 2020) However, it is important to note that without a healthy control group, we cannot definitively establish what constitutes a “normal” level of connectivity between secondary AC and DLFPC. (CC Bauer et al., 2020; Zweerings et al., 2019; Fovet et al., 2016)

These findings tentatively align with models of AHs emphasizing disrupted connectivity between auditory processing and cognitive control regions, (Humpston et al., 2020; Alderson-Day et al., 2016; Collin et al., 2021) suggesting that neurofeedback may potentially reconfigure these functional connections (though our interpretation remains limited by the absence of a healthy control group). Future research with larger sample sizes and long-term symptom tracking is needed to confirm and extend the significance of these findings.

Mental-noting was practiced across Real-NFB and Sham-NFB groups. The reduction in AHs and PAC activation across participants may be attributed to this shared approach, aligning with research showing that mindfulness can modulate sensory processing. (Lutz et al., 2008; Treves et al., 2024; Antonova et al., 2015; Miyashiro et al., 2019) However, non-specific mechanisms like placebo effect or subject expectation may also play a role. (Chiesa et al., 2010) Mental-noting involves labeling present-moment experiences without elaboration, thereby cultivating awareness and non-judgmental observation. (Anālayo, 2003) These aspects may be relevant, as they potentially reduce emotional reactivity and distress associated with AHs. (Chadwick et al., 2009; Lüdtke et al., 2020) The selective engagement of secondary AC and rDLPFC in the Real-NFB group suggests interplay between neurofeedback and mindfulness techniques. While non-specific factors may also influence primary sensory areas, we suspect noting-practice contributed to its modulation (Lutz et al., 2008; Treves et al., 2024; Kerr et al., 2011) irrespective of neurofeedback type; however this needs systematic exploration. Moreover, mental-noting with Real-NFB appears to facilitate specific modulation of secondary AC. (Sherwood M et al., 2018; Wang et al., 2018) Present-moment focus might enhance participants’ engagement with the neurofeedback task, improving attention and reducing mind-wandering. (Treves et al., 2024; Farb et al., 2007; CC Bauer et al., 2020; Mrazek et al., 2012) This combination may represent a novel approach to addressing neural dynamics underlying AHs, providing “top-down” regulation alongside specific neuromodulation. (Tang et al., 2015) This approach of leveraging mindfulness benefits with targeted neuromodulation may offer a more comprehensive treatment strategy for AHs than either alone.

Importantly, our study design did not include a condition with mental-noting practice only, which limits our ability to definitively separate the effects of mindfulness meditation from neurofeedback. A comprehensive design including mental-noting only while in the scanner, in addition to the conditions used in the current study, would be necessary to fully disentangle these effects. The absence of mental-noting-only is a limitation, as we cannot determine whether the observed neural changes result from: 1) the combination of mental-noting with anatomically-targeted NFB, 2) mental-noting practice alone, or 3) synergistic effects of both interventions.

The fact that both groups showed reduced AHs but only the Real-NFB group showed specific neural changes (reduced secondary AC activation and AC-DLPFC connectivity) suggests that while mental-noting may contribute to symptom reduction through non-specific mechanisms, the anatomical specificity of NFB targeting is crucial for inducing particular neural modifications. However, this interpretation is complicated by our differential statistical power across measures. Post-hoc analyses revealed we achieved 75–96 % power for detecting neural changes but only 23–27 % power for behavioral outcomes. This power discrepancy means that while we could reliably detect the large neural effects in the Real-NFB group (effect sizes *d* = 1.43–1.47), we were severely underpowered to detect potentially meaningful behavioral differences between groups.

We acknowledge that this design makes it impossible to determine whether the behavioral improvements resulted from mindfulness practice, neurofeedback, or their combination. The similar PSYRATS reductions in both groups (which must be interpreted cautiously given 23–27 % power) could reflect: 1) genuine effects of mental-noting across both conditions, 2) Type II error masking true between-group differences, or 3) non-specific factors such as expectancy effects. Without proper control conditions and adequate power for behavioral measures, these interpretations remain speculative. Future studies should employ factorial designs with sufficient sample sizes (*n* ≥ 64 per group) to parse the independent and interactive effects of these interventions, which would be essential for optimizing treatment protocols and understanding the mechanisms underlying therapeutic change.

While we focused on therapeutic brain changes related to Real-NFB, the effects of Sham-NFB also should be carefully considered. When feedback signals are incongruent with brain states, these misaligned cues may actively interfere with treatment, creating a nocebo effect. (Arina et al., 2017) This interference aligns with principles of neurofeedback training, which relies on operant conditioning where brain activation patterns are reinforced through contingent feedback signals. (Lüdtke et al., 2020; Hellrung et al., 2022) Studies have shown incongruent feedback can impair learning compared to congruent conditions, (Ossadtchi et al., 2017) suggesting misaligned feedback actively disrupts the neurofeedback learning process. (Arina et al., 2017; Trambaiolli et al., 2021; Cortese et al., 2017) If the goal is to induce neural plasticity through neurofeedback, misleading signals could reinforce maladaptive brain patterns. (Pérez-Elvira et al., 2021) Thus, careful consideration of control conditions that do not interfere with the neurofeedback process is crucial for clinical research, especially among vulnerable populations. (Sorger et al., 2019)

## 5. Limitations

Several limitations warrant consideration. First, the use of different MRI scanners may have introduced hardware variability affecting BOLD signal estimates, though we mitigated this through within-subject longitudinal measurements on the same scanner and balanced cross-condition comparisons across locations. Second, the absence of a double-blind design may have introduced bias, as participants could potentially infer their group assignment.

Most critically, our sample size (*n* = 23) severely constrained statistical power, particularly for behavioral outcomes. Post-hoc power analyses revealed a striking discrepancy: while we achieved moderate to excellent power for neural effects (75.2 % for within-group secondary AC activation, 89.9 % for between-group secondary AC, 91.2 % for AC-rDLPFC connectivity, and 96.0 % for primary AC), we were severely underpowered for behavioral measures (only 8.3 % for between-group PSYRATS comparisons and 22.8–26.8 % for within-group analyses). This suggests our non-significant between-group PSYRATS differences likely reflect insufficient power rather than true equivalence. Further-more, the significant within-group PSYRATS improvements (*p*=.04) should be interpreted cautiously given the low power—they may represent Type I error or indicate our assumed pre-post correlation (*r* =0.5) underestimated the true relationship.

This power discrepancy reveals an important consideration for clinical neurofeedback research: neuroimaging measures appear substantially more sensitive to intervention effects than behavioral symptom scales. While neural markers achieved adequate power even with our small sample, detecting reliable clinical improvements requires much larger samples—approximately 64 participants per group for 80 % power to detect medium behavioral effects (*d* = 0.5). The gradient in neural measure sensitivity (primary AC: 96.0 % > between-group connectivity: 91.2 % > within-group activation: 75.2 %) suggests that different neural outcomes may serve as more or less sensitive biomarkers of treatment response. Additionally, without a mental-noting-only control condition, we cannot isolate neurofeedback-specific effects from meditation-related changes.

Finally, a single neurofeedback session may be insufficient for participants to develop robust self-regulation strategies. Future research should examine optimal session parameters, including duration and frequency, to maximize therapeutic outcomes. Despite these limitations, our well-powered neural findings, particularly the robust between-group differences in connectivity and activation patterns, provide preliminary mechanistic evidence for the therapeutic potential of anatomically-targeted neurofeedback in treating auditory hallucinations.

## 6. Conclusion

This study demonstrates that real-time fMRI neurofeedback combined with mental-noting practice can effectively modulate brain activity and connectivity patterns implicated in AHs. Despite limited statistical power for behavioral outcomes (8.3–26.8 %), we observed robust neural changes with adequate power (75.2–96.0 %), particularly the reduction in functional connectivity between secondary AC and rDLPFC in the Real-NFB group (91.2 % power) and between-group differences in secondary AC activation (89.9 % power). These well-powered neural findings, along with the trend-level relationship between connectivity changes and AHs reduction, provide mechanistic evidence for the therapeutic potential of anatomically-targeted neurofeedback.

Our results highlight both the promise and challenges of combining targeted neurofeedback with mindfulness techniques for treating AHs. The stark contrast between our ability to detect neural changes versus behavioral improvements underscores that while neuroimaging biomarkers may serve as sensitive early indicators of treatment response, demonstrating clinical efficacy requires substantially larger samples. Future studies should employ factorial designs with adequate power (*n* ≥ 64 per group) to disentangle the effects of neurofeedback from mindfulness practice, implement double-blind procedures to minimize bias, and investigate optimal session parameters including duration and frequency. Despite these limitations, the consistency and magnitude of our neural findings suggest that STG-targeted neurofeedback represents a promising avenue for developing precision interventions for treatment-resistant auditory hallucinations.

## Supplementary Material

1

Supplementary material associated with this article can be found, in the online version, at doi:10.1016/j.pscychresns.2025.112050.

## Figures and Tables

**Table 1:** Sociodemographic and clinical information.

	Real condition	Sham condition	Real vs. Sham
*N*	*n* = 10	*n* = 13	
**Sociodemographic**			
Age (mean/SD)	34.6 (10.01)	35.1 (10.65)	*t*-test: *p*=.82
Sex: female (*n*/ %)	2/20 %	4/30.7 %	Wald test: *p*=.57
Race: White (*n*/ %)	6/60 %	11/84.6 %	Wald test: *p*=.19
Education [years] (mean/SD)	13.7 (3.4)	14.0 (2.0)	*t*-test: *p*=.63
IQ: WASI (mean/SD)	99.5 (21.2)	97.3 (16.9)	*t*-test: *p*=.44
**Auditory Verbal Hallucination Severity (mean/SD)**			
PSYRATS			
Baseline	25.16 (6.99)	26.25 (7.08)	*t*-test: *p*=.82
Post-NFB	21.66 (9.0)	22.3(7.31)	*t*-test: *p*=.45
Post-NFB* (*n* = 11)	19.5 (5.0)	22.62 (6.75)	*t*-test: *p*=.52

*Note*. All Real vs. Sham tests were two-tailed independent samples tests. PSY-RATS=The Psychotic Symptom Rating Scales. WASI=Wechsler Abbreviated Scale Intelligence (Full Scale) NFB=real-time fMRI neurofeedback,*NFB=Real-after-Sham-NFB (*n* = 11).
